# Molar incisor hypomineralization, prevalence, pattern and distribution in Sudanese children

**DOI:** 10.1186/s12903-020-01383-1

**Published:** 2021-01-06

**Authors:** Hanna E. Abdalla, Amal H. Abuaffan, Arthur Musakulu Kemoli

**Affiliations:** 1 Department of Orthodontic, Paedodontic and Preventive Dentistry, Faculty of Dentistry, University of Khartoum, P.O. Box 102, Khartoum, Sudan; 2 Department of Paediatric Dentistry and Orthodontocs, University of Nairobi, P.O. Box 34848, Nairobi, 00100 Kenya

**Keywords:** MIH, Prevalence, Pattern, Distribution, Sudanese children

## Abstract

**Background:**

Molar incisor hypomineralization (MIH) has serious impact on oral health-related quality of life for a child, due to its effects on tooth structure, aesthetics and behavior of the child. The current study was designed to determine the prevalence, pattern and distribution of MIH in school children in Sudan.

**Methods:**

This was a descriptive cross-sectional study involving 568 children, aged 8–11 years from schools in Khartoum State. Following the collection of their socio-demographic data, the children were examined for hypomineralization on the 12 MIH-index teeth, the pattern and distribution of the MIH. The data collected was analyzed to obtain descriptive statistics. The results related to the socio-demography and other dental-related factors were tested using chi-square test and Spearman Rank Correlation, with the significant level set at *p* < 0.05.

**Results:**

The prevalence of MIH in the study population was 20.1%. The majority of the participants had both permanent first molars (PFMs) and permanent incisors affected (12.5%). However, in 7.6% of the cases only molars were affected. Even though more maxillary teeth were affected when compared to the mandibular teeth, there was no statistical significant difference between the occurrence of hypomineralization on mandibular and maxillary molars (*p* = 0.22). Maxillary incisors were significantly more affected by MIH when related to the mandibular ones (*p* = 0.00). Demarcated opacities were the commonest pattern of MIH defects (69.9%) in the experimental group.

**Conclusion:**

The prevalence of MIH in Sudanese children was 20.1%. In both dental arches, the permanent molars and incisors were frequently affected, with the demarcated opacity type of MIH being the most common form of defect.

## Background

Molar Incisor Hypomineralization (MIH) is observed on permanent molars as demarcated opacities that vary from creamy-white or yellow to yellowish-brown discoloration. The condition is of systemic origin, affecting one to four permanent first molars (PFMs) and often involving the permanent incisors as well [1]. MIH-like defects have also been detected on the second primary molars, second permanent molars and tips of permanent canines [1–3]. Both the severity of the defects and the number of teeth affected are variable. The yellow/brown defects are considered more severe than the white/creamy opacities [4].

Hypomineralized enamel has higher porosity and lower mechanical resistance that may result in the tooth not just being susceptible to dental caries, but also susceptible to post-eruptive breakdown (PEB) when the affected tooth is under occlusal load [5]. Atypical restorations (AR) involving uncommon surfaces of teeth like cusps can be noticed with irregular margins and opacity around the restorations. Children with MIH may experience severe tooth sensitivity to temperature changes making it difficult to maintain oral hygiene and further increasing the caries risk [6]. Hypomineralization in incisors presented mainly as demarcated opacities affecting one third of the incisal part and has major esthetic concerns. While in molars, the opacities affect one third of the occlusal surface and usually involve the cusps; this can lead to PEB, dental caries and extraction due to MIH [5].

**Fig. 1:**
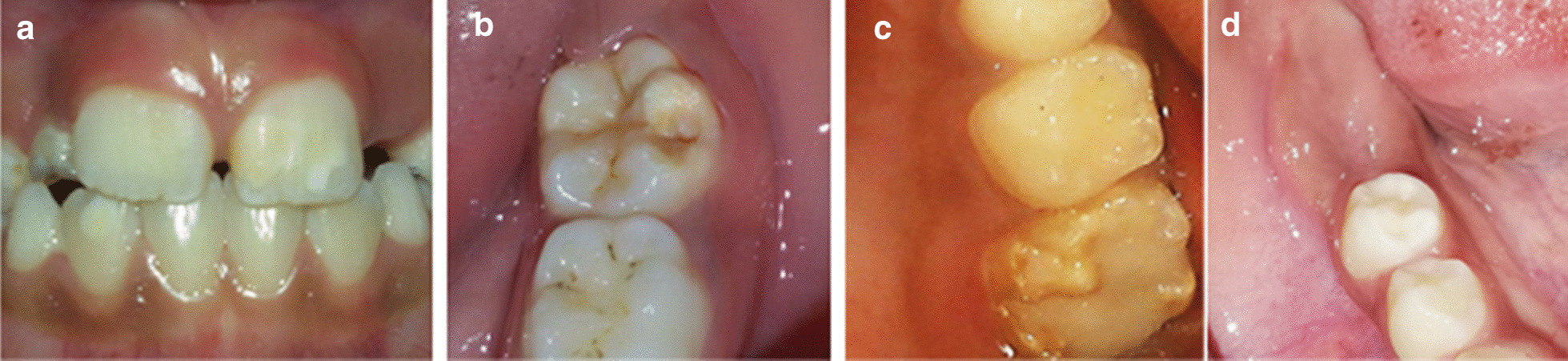
Photographs taken during the study showing MIH diagnosis as based on the European Academy of Paediatric Dentistry (EAPD) diagnostic criteria: **a** Demarcated enamel opacities**, b** post-eruptive breakdown, **c** atypical restoration (note the irregular margins extended to palatal surface and opacity at the border of the restoration), and **d** extraction because of MIH

The European Academy of Paediatric Dentistry (EAPD) in 2003 developed a diagnostic criteria of MIH that include demarcated opacities, post-eruptive enamel breakdown, atypical restorations and extraction due to MIH [5] as features to observe (Fig. 1). The diagnosis of MIH is clinically determined while the tooth is clean and wet, to distinguish it from conditions that can mimic it like enamel hypoplasia, fluorosis, amelogenesis imperfecta and early carious lesions. However, in hypoplasia, the borders of the deficient enamel are distinct, while for the MIH lesions, the borders of enamel are irregular [7]. In the case of fluorosis, the enamel opacities are diffused unlike the demarcated borders of MIH, while in amelogenesis imperfecta the defects are generalized and usually there is a family history [7].

The etiology of MIH is unknown, but environmental factors like child delivery complications, premature birth, dioxins in breast milk, respiratory problems, calcium and phosphate metabolic disorders, high fevers during early childhood and use of antibiotics; as well as genetic factors, have been linked to this condition [8, 9]. What has been clear about MIH is that the offending factors come into play during the first four years after birth, and interfere with the calcification and/or maturation phases of amelogenesis resulting in qualitative defects of enamel or hypomineralization [10].

MIH is currently more frequently seen in dental clinics which represents a big challenge for dental practitioners. Management modalities for teeth with MIH may range from prevention, restoration and even extraction. Long-term treatment concepts have included desensitizing and remineralizing  products, resin infiltration, sealants, micro-abrasion, composites, veneers and crowns [11].

Globally, the prevalence of MIH has been estimated to range from 2.4 to 40.2% [12] with an estimated mean of 13.1 to 14.2% [13, 14]. In Africa, the few available studies have shown the prevalence of MIH to range from 2.3 to 17.7% [15–18]. As there are no past studies on MIH reported from Sudan, this study was designed to determine the prevalence, clinical pattern and distribution of MIH in a group of Sudanese children. The null hypothesis was that the prevalence and distribution of MIH in Sudanese children does not differ from the mean global prevalence, irrespective of age, gender or dental arches.

## Methods

This was a descriptive, cross-sectional study undertaken in 2017, involving 8–11 year-old children from public basic schools in Khartoum state.

### Determination of study population

The study sample was determined using the formula, $n = \frac{{z^{2} pq}}{{\left( {d^{2} } \right)}}deff$ with n = sample size, *z* = critical value for achieving 95% confidence level, *p* = the anticipated population proportion which is always chosen from previous studies,

*q* = *1-p*, *d* = desired margin of error and *deff* = design effect chosen as 2.

As for *p* value, a Kenyan study of 2009 that reported MIH prevalence of 13.73% [16] was used. Assuming an error of 4% in applying the formula, a sample size of 568 children was arrived at.

### Sampling technique

Four localities out of the seven Localities forming Khartoum State were randomly selected, and through a multi-stage cluster sampling technique. Twenty basic schools were randomly selected proportionally to the child population in the selected localities. Ethical approval for the study was obtained from the Research Committee of University of Khartoum—Faculty of Dentistry (HREC-5/2015), besides receiving written approvals from the Director of primary school education for each locality and the Director of each primary schools in Sudan. A written informed consent was obtained from the parents/guardians of the participants, and each child also gave assent to participate in the study.

### Inclusion criteria

Sudanese Children aged 8–11 years old, attending the primary schools in Khartoum State and having their MIH index teeth erupted (i.e. permanent first molars and incisors).

### Exclusion criteria

Children who had enamel lesions smaller than 2 mms [12], those with other enamel defects (e.g. fluorosis and hypoplasia) and those who refused to participate or absent from the school at the time of study.

### Data collection

The Principal Investigator was initially trained by an experienced paediatric dentist on MIH detection. The training involved the use of photographs and later the actual examination of children under field conditions who were not part of the study participants. During this phase and during the time of data collection, inter-examiner calibration was done and Kappa values calculated, with mean value of 0.83. The Principal Investigator also re-examined every tenth participant to determine intra-examiner value, which was calculated as 0.84.

In carrying out the study, the Principal Investigator was assisted by a trained and pre-tested recording clerk during the examination for the MIH of the 8–11 years-old children. Participants were selected from third, fourth and fifth classes from the 20 schools. A total of 640 consent forms were sent to the eligible children’s parents. From which, 23 were not signed. All the children whose parents consented were included in the study. Nonetheless, 49 of them were excluded for various reasons, like absence from the school on the day of examination and those who had other enamel lesions (e.g. fluorosis and hypoplasia). Consequently, a total of 568 children (284boys,284girls) were included in the study. Socio-demographic data of each participant were first recorded using a modified World Health Organization oral health assessment form for children (2003) including child’s name, gender, age, locality and school level, prior to the documentation of the findings of the oral examination.

The oral examination took place in a room prepared for the purpose, in the respective schools of the participants. Each child was examined while sitting in up-right position in an ordinary chair facing a natural light source. The examiner used sterile mirrors, dental probes, tweezers, cotton rolls, in addition to single use of clean disposable examination gloves and face masks for each child. The probe was initially used to gently remove dental plaque and food remnants from the tooth surfaces. Cotton rolls were used to clean the teeth surfaces prior to examining them for MIH.

The index teeth (i.e. permanent first molars and incisors) for each participant were examined, while wet; for the presence of demarcated opacities, post-eruptive enamel breakdown, atypical restorations (AR) and extraction due to MIH [7]. Hypomineralization defects were recorded in accordance with EAPD scoring criteria for MIH [19]. Children were considered as having MIH when one or more PFMs were affected, with or without the involvement of incisors. Opacities occurring in permanent incisors but not in at least one PFM were not recorded as MIH. All children in the participating schools received free oral health education and the study participants who required dental treatment were referred for appropriate management to the Pediatric Department, Faculty of Dentistry/University of Khartoum.

### Data analysis

Data collected were analyzed using Statistical Package for Social Science (SPSS) computer program Version 19. Descriptive data like frequency, means and relative distributions of MIH were displayed using Tables and Bar Charts. The Chi-square test was used to test the association between MIH and age, gender; the difference in proportions between two groups like molars and incisors, left and right, maxillary and mandibular teeth. Spearman rank correlation was used to test the association between the number of affected molars and incisors. In all these tests the p value was pegged at < 0.05, which was considered to be significant.

## Results

A total of 568 (284 boys, 284 girls) children aged 8- to 11-year-old (with mean age = 9.5) were examined for MIH. There were no significant statistical differences noted between the participants’ ages or gender in relation to the occurrence of MIH (*p* > 0.05) (Table 1).

**Table 1:** Distribution of MIH among different ages and gender

Variables	Children with teeth with MIH (%)	Children with teeth without MIH (%)	Total number of children (%)	Chi-square	*p* value
*Gender*					
Boys	59 (20.7%)	225 (79.3%)	284 (50%)	0.176	0.675
Girls	55 (19.4%)	229 (80.6%)	284 (50%)		
Total	114 (20.1)	454 (79.9)	568 (100%)		
*Age in years*					
8	18 (19.6%)	74 (80.4%)	92 (16.2%)	4.297	0.231
9	29 (16.1%)	151 (83.9%)	180 (31.7%)		
10	43 (20.9%)	163 (79.1%)	206 (36.3%)		
11	24 (26.7%)	66 (73.3%)	90 (15.8%)		

No statistically significant difference was found (*p* > 0.05)

MIH, molar incisor hypomineralization

### Prevalence of MIH

MIH was observed in 114 of the 568 children examined, giving a prevalence of 20.1% (95% CI 16.7–23.3). Of the 114 participants with MIH, 71 (12.5%) had hypomineralization changes in their permanent first molars (PFM) and permanent incisors (PI), 43 (7.6%) participants had molar hypomineralization (MH). Incisor hypomineralization (IH) was found in 18 children (3.2%) of the population (not MIH). The total numbers of affected teeth for children with MIH were 376 with a mean of 3.3 (± 1.63) teeth per child, of which 2.2 and 1.1 teeth were molars and incisors respectively. There was no significant statistical difference in the mean number of affected teeth between boys and girls (*p* = 0.386).

### Distribution of MIH affected teeth

In terms of the distribution of MIH defects on the MIH index teeth, the majority of the children (38 or 33.3%) had one molar affected, 34 (29.8%) had 2 molars, 27 (23.7%) had 3 molars while only 15 (13.2%) of the children had all the four molars affected. The mean number of affected incisors increased with increasing number of affected molars, and the difference was statistically significant (*p* value < 0.001) (Table 2). The details for specific teeth affected by MIH are provided in Table 3. The PFMs had a significantly higher rate of occurrence of MIH (65.8%) than the incisors (34.2%). However, there was no significant statistical difference in MIH occurring on the right (50.5%) and left side (49.5%) of the dental arches (*p* = 0.837).

**Table 2:** Mean number of affected permanent incisors according to the number of affected PFMs

No. of molars affected	Mean no. of incisors affected (95% CI)	Spearman rank correlation	*p* value
1	1.16 (0.79–1.53)	0.627	< 0.001^a^
2	0.92 (0.58–1.25)		
3	1.07 (0.61–1.54)		
4	1.67 (0.81–2.52)		

No, number; CI, confidence interval

^a^Statistically significant

**Table 3:** Distribution of hypomineralization features in index teeth of MIH-affected children according to EAPD diagnostic criteria (2003)

Index tooth	White/creamy demarcated opacities with no PEB N (%)	White/creamy demarcated opacities with PEB N (%)	Yellow/brown demarcated opacities with no PEB N (%)	Yellow/brown demarcated opacities with PEB N (%)	Atypical restorations N (%)	Missing due to MIH N (%)	Total N (%)	Chi square	*p* value
16	32 (13.6%)	15 (20.3%)	8 (28.6%)	7 (22.6%)	0 (0%)	0 (0%)	62 (16.5%)	200.80	< 0.001^a^
12	4 (1.7%)	0 (0%)	0 (0%)	0 (0%)	0 (0%)	0 (0%)	4 (1.1%)	NA	NA
11	39 (16.6%)	2 (2.7%)	0 (0%)	1 (3.2%)	0 (0%)	0 (0%)	42 (11.2%)	170.3	< 0.001^a^
21	32 (13.6%)	1 (1.4%)	0 (0%)	1 (3.2%)	0 (0%)	0 (0%)	34 (9.0%)	174.7	< 0.001^a^
22	7 (3%)	0 (0%)	0 (0%)	0 (0%)	0 (0%)	0 (0%)	7 (1.9%)	215.4	< 0.001^a^
26	32 (13.6%)	10 (13.5%)	8 (28.6%)	6 (19.4%)	0 (0%)	0 (0%)	56 (14.9%)	289.52	< 0.001^a^
36	26 (11.1%)	16 (21.6%)	6 (21.4%)	12 (38.7%)	1 (33.3%)	2 (40%)	63 (16.8%)	102.59	< 0.001^a^
32	9 (3.8%)	0 (0%)	1 (3.6%)	0 (0%)	0 (0%)	0 (0%)	10 (2.7%)	NA	NA
31	9 (3.8%)	0 (0%)	0 (0%)	0 (0%)	0 (0%)	0 (0%)	9 (2.4%)	511.03	< 0.001^a^
41	8 (3.4%)	0 (0%)	0 (0%)	0 (0%)	0 (0%)	0 (0%)	8 (2.1%)	105.98	< 0.001^a^
42	14 (6.0%)	0 (0%)	1 (3.6%)	0 (0%)	0 (0%)	0 (0%)	15 (4%)	294.6	< 0.001^a^
46	23 (9.8%)	30 (40.5%)	4 (14.3%)	4 (12.9%)	2 (66.7%)	3 (60%)	66 (17.6%)	215.4	< 0.001^a^
Total	235 (100%)	74 (100%)	28 (100%)	31 (100%)	3 (100%)	5 (100%)	376 (100%)	86.0	< 0.001^a^

N, number; NA, not applicable; MIH, molar incisor hypomineralization; PEB, Post-eruptive breakdown

^a^Highly significant differences were noted (*p* < 0.05)

In general, the maxillary teeth were more affected (55.5%) when compared to the mandibular, and this difference was statistically significant (*p* = 0.017). However, when only the molars were evaluated, the mandibular molars were slightly more affected than the maxillary molars, and the difference was not statistically significant (*p* = 0.218). For the permanent incisors, the maxillary incisors were found to be significantly more affected than the mandibular incisors (*p* = 0.00). The central incisors in the upper jaw were significantly more affected than the lateral incisors (*p* = 0.000), while in the lower jaw the lateral incisors were more affected than the central incisors with the difference not being significant statistically (*p* = 0.103). Overall, the most frequently affected tooth was the left mandibular PFM (17.6%); while, the least was the right maxillary lateral incisor (1.1%).

### Pattern of the MIH defects

**Fig. 2:**
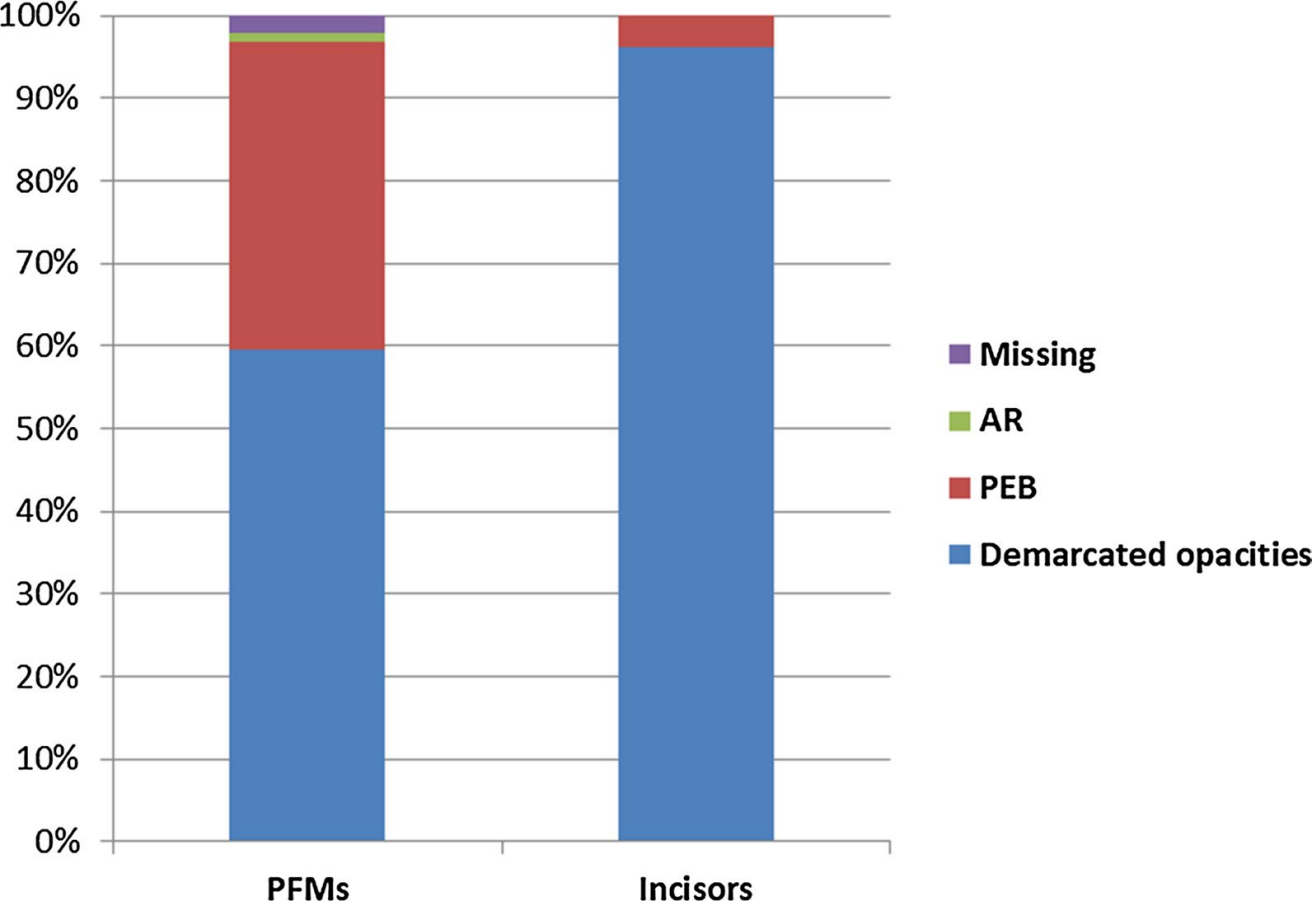
Pattern of MIH defect of hypomineralized teeth, showing that demarcated opacities is the commonest defect in both permanent first molars (PFMs) and incisors, Posteruptive breakdown (PEB) was higher in PFMs than incisors and no cases of atypical restorations (AR) or missing because of MIH in incisors

Of the 376 scored MIH-affected PFMs and incisors teeth, the demarcated opacities were the most common pattern of defect (69.9%) followed by PEB (28%). Atypical restorations and extractions were present among the PFMs only; their frequency was (0.8%) and (1.3%) respectively (Table 3). The white/creamy discolorations were more frequent than the yellow brown demarcated opacities. Figure 2 shows the pattern of MIH defects for each group of index teeth. Overall, post-eruptive breakdown was more frequent in girls than boys, but the difference was not significant statistically (*p* = 0.662), neither was there a statistical significant difference in relation with the age of the participants (*p* = 0.598) (Table 4).

**Table 4:** Post-eruptive breakdown frequency in terms of age and gender

Variables	White/creamy demarcated opacities with PEB	Yellow/brown demarcated opacities with PEB	Total	Chi-square	*p* value
*Gender*					
Boys	30	14	44	0.192	0.662
Girls	44	17	65		
Total	74	31	105		
*Age in years*					
8	10	2	12	1.877	0.598
9	13	8	21		
10	28	11	39		
11	22	11	33		
Total	73	32	105		

No statistically significant difference was found (*p* > 0.05)

PEB: Post-eruptive breakdown

## Discussion

This was a field epidemiological study on the prevalence of MIH in Sudanese children aged 8–11 years, where 20.1% of the participants were diagnosed with MIH. The majority of the affected teeth were in the maxillary arch. The Null hypothesis was disproved in the case of MIH prevalence and distribution, but proved in the case of correlation of MIH with age and gender. The EAPD MIH index was used for the diagnosis of MIH, because it is easy for clinicians to record, and has also been tested in other studies [20, 21]. Only opacities of 2 mm and larger were included because enamel lesions less than 2 mm are quite common [12]. Although the age of 8 years is recommended for studies dealing with MIH, however, in this study we included up to the age of 11 years. This was anticipated to include more participants and also to enable detection of more patterns of the defects as described in the study, besides other studies have also used age groups higher than 8 years [22].

### Prevalence of MIH

The prevalence of MIH in the current study was 20.1%, which was higher than the mean global prevalence (13.1% to 14.2%) reported in 2018 [13, 14], but comparable to results reported in Spain (21.8%) [23], Japan (19.8%) [24], Thailand (20.0%) [25], Iran (20.2%) [26] and Iraq (21.5%) [19]. However, our results were found to be lower than those reported in Lebanon (26.7%) [27] and Dubai (27.2%) [28] but higher than those reported from Nigeria (17.7%) [17], Kenya (13.7%) [16], Libya (2.9%) [15] and Egypt (2.3%) [18]. There was no difference in the occurrence of MIH with age in this study, in line with results reported by Oydele et al. [17] and Ghanim et al., [29] but in contradiction with studies that showed significant increase with age by Da costa-Silva et al. [30] and the decrease of MIH with age by Saitoh et al. [24]. In terms of gender, the current study showed boys had slightly higher MIH prevalence than girls, although this was not statistically significant, just as some studies have indicated before [24, 27] but contrary to results from other studies where the prevalence were higher for girls [16] and for boys [31].

### Number and distribution of MIH-affected teeth

The mean number of MIH-affected teeth per child in the current study was 3.3, consistent with that reported in Brazil of 3.3 [20], Nigeria with 3.5 [32] and India with 4.31 [31]. The consistency in the mean number of affected teeth could suggest that regardless of the MIH prevalence in the population, the mean number of teeth affected appear to be almost similar, a denotation of a common characteristic of the defect.

The majority of the children in this study had one and two molars affected rather than three or four molars, the same finding as reported in a study in Ankara, Turkey [33] but in contrast with other studies that have reported most of children having all the four molars affected [29]. The risk of incisor involvement appeared to increase significantly with the number of molars affected, a situation similar to the findings in a study conducted by Da costa-Silva et al. [30] but in contrast to that found that the risk was insignificant [23, 34].

In the current study, the maxillary teeth affected by MIH were more than the mandibular teeth, a situation consistent with the findings by Temilola et al. in 2015 [32], but studies by Ghanim et al. in 2011 [19] and Elzein et al. [27] found equal distribution of MIH defects in the upper and lower jaws. The current study also showed that the mandibular and maxillary molars were equally affected in contradiction to the results from Jordan [21] and Gujarat, India [35] with more mandibular molars affected and an Iraqi study with more maxillary molars affected [19]. Furthermore, the maxillary incisors in the current study were more frequently affected than the mandibular incisors, a result that mirrored the findings by Parikh et al. in 2012 [35] and Ghanim et al. in 2011 [19], but contrary to the results by Sönmez et al. in 2013 [33] who reported more mandibular than maxillary incisors being affected.

### Pattern of MIH defect in hypomineralized teeth

In this study, demarcated opacities were the most frequent patterns amongst the affected teeth. This finding was similar to the results by Ahmadi et al. [26] and Elzein et al. [27]. The white/creamy demarcated opacities were more frequent than yellow/brown opacities, which was in agreement with previous study by Mittal et al. in 2015 [36] but just the opposite of the findings by Ghanim et al. in 2014 [29]; which revealed that yellow/brown opacities were the most common form of MIH defects. The prevalence of PEB in the present study was 28% which was comparable to results obtained by Allazzam et al. (26.1%) [34]. This finding may be explained by the inclusion of older age group children in the current study, as demarcated opacities may tend to break down over time.

## Conclusions and recommendations

The Findings from this study show the following:

- The prevalence of MIH in Sudanese school children from Khartoum State was 20%, with no significant gender predilection. The children in the study had both their permanent first molars and permanent incisors frequently affected by MIH defects. However, whereas the mandibular PFMs were more frequently affected by MIH than the maxillary PFMs, the maxillary incisors were significantly more affected than mandibular incisors.
- The most prevalent form of MIH defect noted in the study was the demarcated opacities type followed by post-eruptive breakdown, and the least common type of MIH was the atypical restorations and missing because of MIH.
- The current study has shown a need for provision of education on diagnosis and management of MIH to all dental practitioners in Sudan to help them on early recognition and appropriate management of this condition.
- Further studies are required for in-depth analysis on aetiological factors associated with MIH, treatment approaches and impact of MIH on oral health-related quality of life of children in Sudan.

## Limitations of the study

- Diagnosis of MIH was made by inspection under the daylight. This may have resulted in the examiner missing some cases of MIH and possible confusion with other enamel defects.
- Many other socio-demographic data were not recorded, such as residence, place of birth, parents’ education and income.

Despite these limitations, the study provides useful information on the situation of MIH in Sudan, and the need for more studies on this subject to enlighten the clinicians on the severity of MIH in children in Sudan.

## Data Availability

The data provided for the results presented in this study is available through the corresponding author, but restrictions apply to the availability of these data and to a certain time period, as the data were used under license for the current study, and so are not publicly available.

## Abbreviations

AR: Atypical restoration
IH: Incisor hypomineralization
MH: Molar hypomineralization
MIH: Molar incisor hypomineralization
PEB: Post eruption breakdown
PFM: Permanent first molars
PI: Permanent incisors
SPSS: Statistical Package for Social Sciences
